# Genome mining reveals novel biosynthetic gene clusters in entomopathogenic bacteria

**DOI:** 10.1038/s41598-023-47121-9

**Published:** 2023-11-25

**Authors:** Wipanee Meesil, Paramaporn Muangpat, Sutthirat Sitthisak, Triwit Rattanarojpong, Narisara Chantratita, Ricardo A. R. Machado, Yi-Ming Shi, Helge B. Bode, Apichat Vitta, Aunchalee Thanwisai

**Affiliations:** 1 Department of Microbiology and Parasitology, Faculty of Medical Science, Naresuan University, Phitsanulok, 65000 Thailand; 2 Centre of Excellence in Medical Biotechnology (CEMB), Faculty of Medical Science, Naresuan University, Phitsanulok, 65000 Thailand; 3 Department of Microbiology, Faculty of Science, King Mongkut’s University of Technology Thonburi (KMUTT), Bangkok, 10400 Thailand; 4 Department of Microbiology and Immunology, Faculty of Tropical Medicine, Mahidol University, Bangkok, 10400 Thailand; 5 Experimental Biology Research Group, Institute of Biology, University of Neuchâtel, Rue Emile-Argand 11, 2000 Neuchâtel, Switzerland; 6 Department of Natural Products in Organismic Interactions, Max Planck Institute for Terrestrial Microbiology, 35043 Marburg, Germany; 7 Molecular Biotechnology, Department of Biosciences, Goethe University, Frankfurt, 60438 Frankfurt am Main, Germany; 8 CAS Key Laboratory of Quantitative Engineering Biology, Shenzhen Institute of Synthetic Biology, Shenzhen Institute of Advanced Technology, Chinese Academy of Sciences, Shenzhen, 518055 China; 9 Chemical Biology, Department of Chemistry, Philipps University Marburg, 35032 Marburg, Germany; 10 Senckenberg Gesellschaft für Naturforschung, Frankfurt am Main, Germany; 11 SYNMIKRO (Zentrum für Synthetische Mikrobiologie), 35032 Marburg, Germany; 12 Center of Excellence for Biodiversity, Faculty of Sciences, Naresuan University, Phitsanulok, 65000 Thailand

**Keywords:** Microbiology, Molecular biology

## Abstract

The discovery of novel bioactive compounds produced by microorganisms holds significant potential for the development of therapeutics and agrochemicals. In this study, we conducted genome mining to explore the biosynthetic potential of entomopathogenic bacteria belonging to the genera *Xenorhabdus* and *Photorhabdus*. By utilizing next-generation sequencing and bioinformatics tools, we identified novel biosynthetic gene clusters (BGCs) in the genomes of the bacteria, specifically *plu00736* and *plu00747*. These clusters were identified as unidentified non-ribosomal peptide synthetase (NRPS) and unidentified type I polyketide synthase (T1PKS) clusters. These BGCs exhibited unique genetic architecture and encoded several putative enzymes and regulatory elements, suggesting its involvement in the synthesis of bioactive secondary metabolites. Furthermore, comparative genome analysis revealed that these BGCs were distinct from previously characterized gene clusters, indicating the potential for the production of novel compounds. Our findings highlighted the importance of genome mining as a powerful approach for the discovery of biosynthetic gene clusters and the identification of novel bioactive compounds. Further investigations involving expression studies and functional characterization of the identified BGCs will provide valuable insights into the biosynthesis and potential applications of these bioactive compounds.

## Introduction

Microorganisms, particularly bacteria and fungi, have played a crucial role in the discovery and development of numerous drugs used in human medicine^1^. Approximately 70–80% of important antibiotics currently in use are derived from microorganisms^2^. For instance, penicillin, streptomycin, tetracycline, erythromycin, and vancomycin, were initially discovered from microorganisms. Furthermore, microorganisms have been instrumental in the discovery of other types of drugs beyond antibiotics. For example, the immunosuppressant drug cyclosporine, the antifungal agent amphotericin B, the antiviral drug acyclovir, and the antiparasitic drug ivermectin are other notable examples also derived from microorganisms^3–6^.

In recent years, there has been growing interest in exploring microorganisms, including bacteria and fungi as potential sources of novel drug candidates. Among the bacteria, *Xenorhabdus* and *Photorhabdus* bacteria have been valuable sources for the discovery of novel antibiotics and other bioactive compounds. These bacteria, in association with entomopathogenic nematodes, produce a wide range of natural products. For instance, *Xenorhabdus* spp. produce xenematides^7^ and xenorxides^8^, which have proven effective against several pathogenic bacteria. Similarly, *Photorhabdus* spp. are recognized for their production of multiple antibiotics, including xenorhabdins—a class of toxic macrolide compounds that exhibit robust antimicrobial properties against both Gram-positive and Gram-negative bacteria^7,9^.

The discovery process often involves screening extracts or purified compounds from these bacteria against panels of bacteria and fungi, including drug-resistant strains. However, advanced techniques like genome sequencing have recently demonstrated that the chemical diversity captured by the traditional culture-based approaches is only the tip of the iceberg, and there are still a significant number of silent or cryptic biosynthetic gene clusters (BGCs), which are not expressed under standard laboratory conditions. Therefore, genome sequencing can be a powerful tool to uncover potential antibiotic candidates, understand the genetic basis of antibiotic production, and guide the discovery and development of novel antibiotics. In this study, we focused on observing BGCs and comparing the of *Xenorhabdus* and *Photorhabdus* bacterial genomes from Thailand. Then we can pinpoint genetic variations and potential candidates responsible for novel antibiotic synthesis.

## Results

### Genome mining of secondary metabolites and BGCs distribution

**Figure 1:**
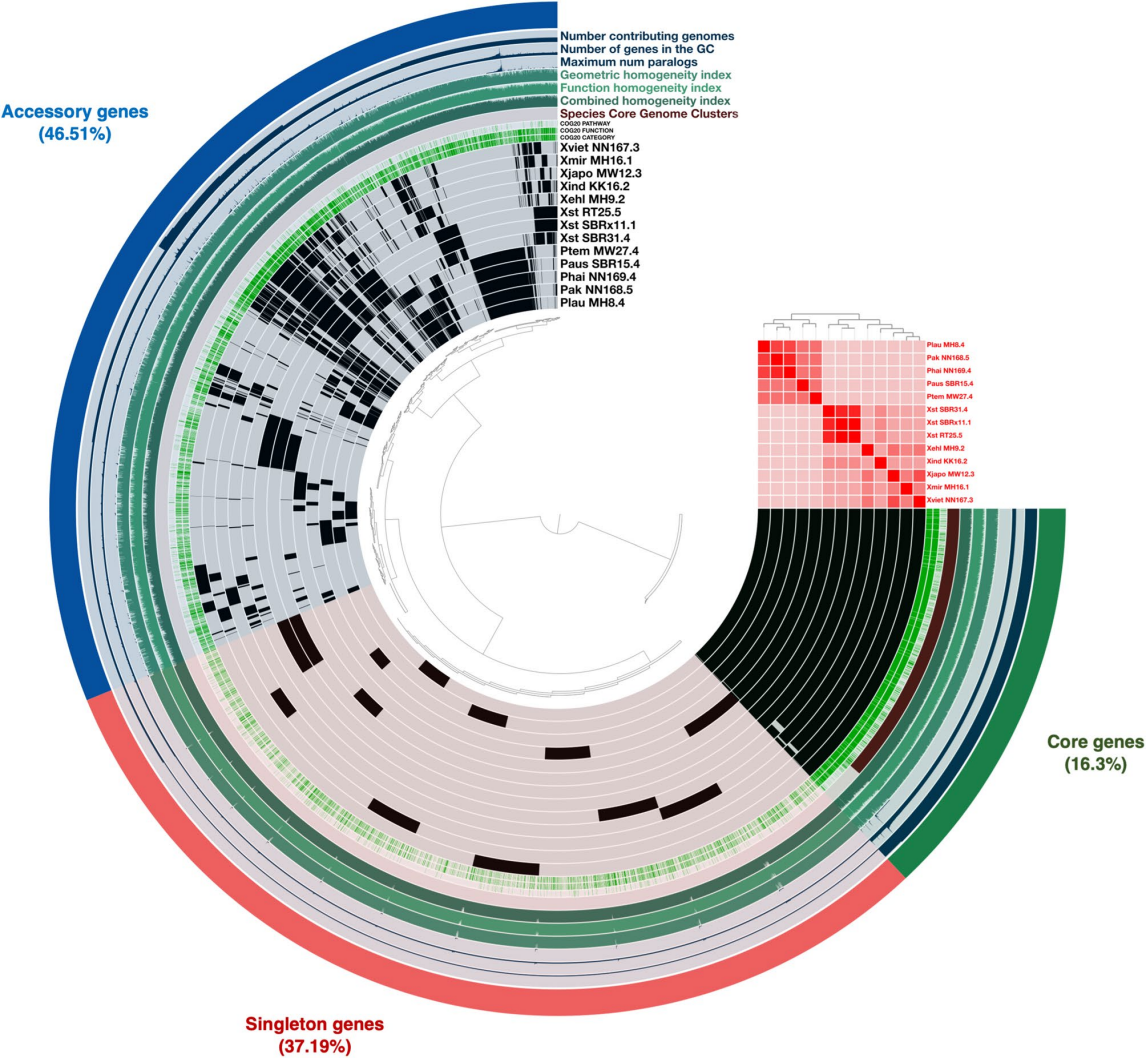
Pan-genome analysis with an average nucleotide identity layer between the bacterial strains. The circular dendrogram was constructed based on the presence or absence of gene clusters. At the upper right corner, average nucleotide identity heatmap are displayed. Each circle represents an individual genome, encompassing all genes (black) from the 13 bacterial genomes. Moving from the outermost layer to inner layers, the core genes (green), accessory genes (blue), and singleton genes (red) were shown. Other information included in the figure comprises the number of contributing genomes, the number of genes in the GC (gene cluster), maximum number of paralogs, geometric homogeneity index, function homogeneity index, combined homogeneity index, Species Core Genome (SCG) clusters, cluster of orthologous groups (COG20) categories, COG20 function, and COG20 pathway.

Combining genetic and biosynthetic diversities may provide insight into how these entomopathogenic bacteria can be prioritized in order to uncover novel chemotypes without redundancy of examinations. The processes were initiated with pan-genome analysis, which illustrated the complete set of genes, containing sequences shared by all individuals (core genes) and those that were either shared among specific individuals (accessory genes) or unique to them (singleton genes). The results of this analysis revealed a total of 51,883 genes across the 13 genomes of *Xenorhabdus* and *Photorhabdus* bacteria (Supplementary Table S1), covering 10,821 gene clusters, which comprised of 1763 core genes, 5033 accessory genes, and 4024 singleton genes. This step help researchers understand the genetic diversity, evolutionary relationships, and functional capabilities of the strains within the group. Likewise, within the cluster of orthologous groups of proteins (COG20) category, there were a total of 6381 known clusters and 4440 unknown clusters. The COG20 function analysis revealed 6383 known clusters and 4438 unknown clusters, while the COG20 pathway analysis identified 1354 known clusters and 9467 unknown clusters (Fig. 1; Supplementary Table S2, and Supplementary Table S3). Subsequently, an antiSMASH software was employed for the preliminary assessment of the bacterial BGCs. The results shown a total of 314 BGCs across 13 genomes of *Xenorhabdus* and *Photorhabdus* bacteria (Supplementary Table S4). The highest biosynthetic diversities were found in *X. indica* strain KK26.2 (Xind KK26.2) and *X. vietnamensis* strain NN167.3 (Xvie NN167.3) followed by *P. temperata* strain MW27.4 (Ptem MW27.4), *P. akhurstii* strain NN168.5 (Pak NN168.5), *P. hainanensis* strain NN169.4 (Phai NN169.4), *P. laumondii* strain MH8.4 (Plau MH8.4), *X. miraniensis* strain MH16.1 (Xmir MH16.1), and *X. ehlersii* strain MH9.2 (Xehl MH9.2). The lowest diversity of BGCs was *P. australis* strain SBR15.4 (Paus SBR15.4), *X. stockiae* strain RT25.5 (Xsto RT25.5), *X. stockiae* strain SBRx11.1 (Xsto SBRx11.1), *X. japonica* strain MW12.3 (Xjap MW12.3), and *X. stockiae* strain SBR31.4 (Xsto SBR31.4). According to the findings above, the average genome size and BGCs abundance of these entomopathogenic bacteria were 4.6 Mb and 24 BGCs, respectively (Table 1). The standard deviations for these values were calculated to be 562,733.6929 and 3.789323734.

**Table 1:** The overall number of predicted genes and BGCs across 13 genomes.

Strain number	Bacteria species	Estimated genome size	Number of predicted genes	Number of predicted BGCs by antiSMASH
Pak NN168.5	*P. akhurstii*	5,629,344	4805	26
Paus SBR15.4	*P. australis*	4,699,665	4152	19
Phai NN169.4	*P. hainanensis*	5,260,969	4337	24
Plau MH8.4	*P. laumondii*	4,937,123	4351	23
Ptem MW27.4	*P. temperata*	5,247,567	4582	26
Xehl MH9.2	*X. ehlersii*	3,916,271	3383	20
Xind KK26.2	*X. indica*	4,506,415	3907	28
Xjap MW12.3	*X. japonica*	3,495,382	3008	18
Xmir MH16.1	*X. miraniensis*	4,378,409	3739	21
Xsto RT25.5	*X. stockiae*	4,509,455	3921	19
Xsto SBR31.4	*X. stockiae*	4,496,578	3832	18
Xsto SBRx11.1	*X. stockiae*	4,509,288	3919	19
Xvie NN167.3	*X. vietnamensis*	4,654,759	3947	28

### Sequence-based similarity network of BGCs

**Figure 2:**
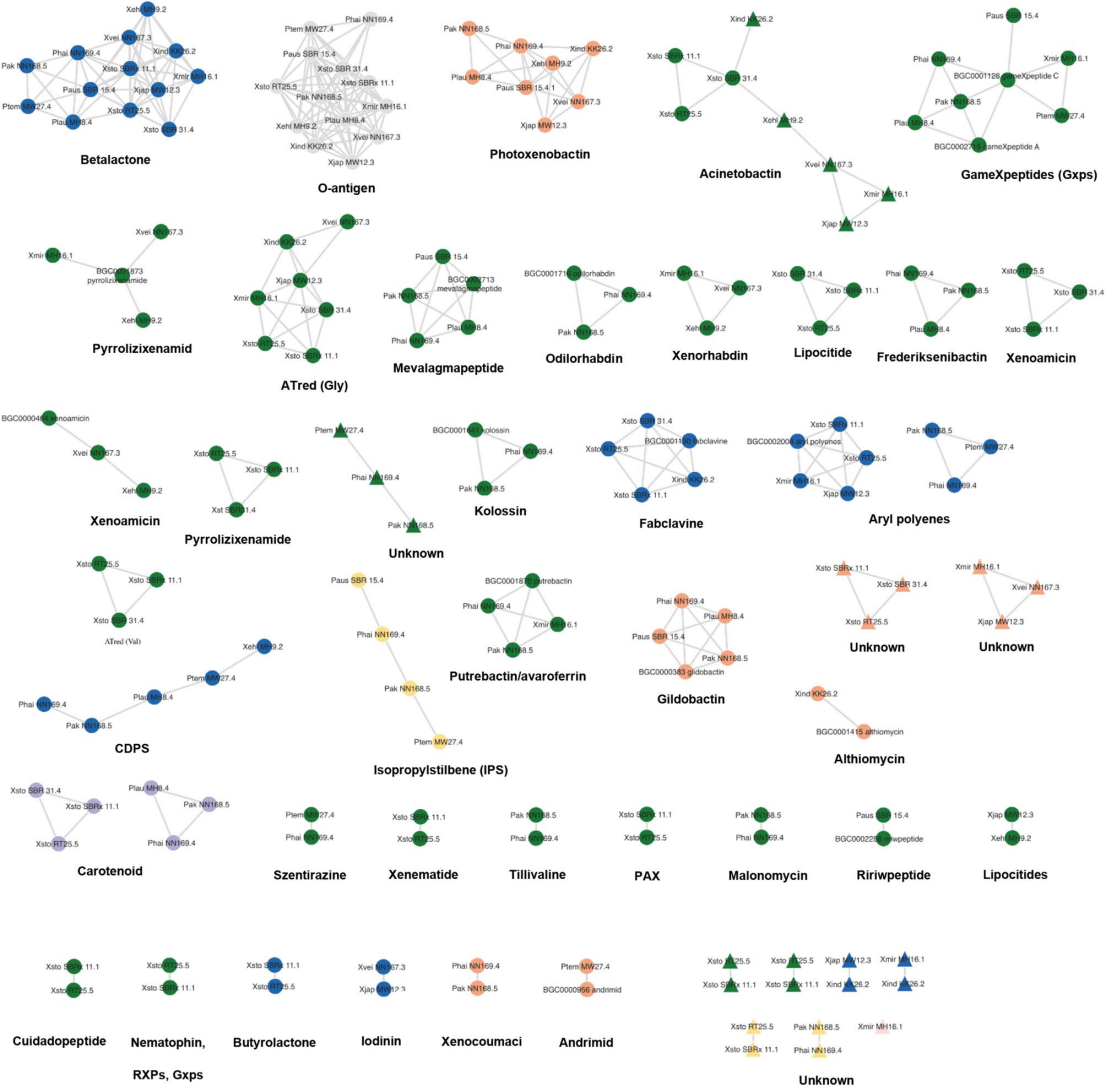
Sequence-based similarity network of biosynthetic gene cluster (BGCs) across 13 genomes of *Xenorhabdus* and *Photorhabdus* bacteria. The network illustrates identified BGCs, denoted by circular shapes for known BGCs and triangular shapes for unknown BGCs. These clusters are categorized by type, including NRPS (green), PKS (yellow), hybrid (orange), RiPPs (gray), terpene (purple), and others (blue).

Although the program antiSMASH is the most employed bioinformatic tool for finding and evaluating BGCs in the genome sequences^10^, it is important to note that the structural information of natural products from antiSMASH is occasionally insufficient. Whilst the technique may assess the substrate specificities of NRPS (Non-ribosomal peptide synthetase) or PKS (Polyketide synthase) modules, it does not take nonlinearity, module-skipping, cyclization, or alterations into account. Despite this, the output from antiSMASH and its embedded tools contains an extensive data for structural information^11^. To assess more accurate biosynthetic abundance, we conducted a network analysis using a combination of bioinformatics tools, including antiSMASH, BiG-SCAPE (Biosynthetic Genes Similarity Clustering and Prospecting Engine)^12^, and manual verification with our in-house database. Here we reported evidence of a relatively high abundance of 178 putative BGCs from 13 genomes of *Xenorhabdus* and *Photorhabdus* bacteria including 89 NRPS, 9 PKS, 22 hybrids, 6 Terpenes, 15 RiPPs (Ribosomally synthesized and post-translationally modified peptides), and 37 others as depicted in Fig. 2 and Supplementary Table S5. Out of the 178 potential biosynthetic clusters, 10 clusters were excluded from the network analysis because insufficient information from the predicted modules. Among the remaining clusters, 146 clusters shown similarity with known BGCs, while 22 clusters represented orphan BGCs for which no known homologous gene clusters could be identified. This suggests the potential novelty of the metabolites associated with these clusters.

### Comparison of the biosynthetic gene clusters

**Figure 3:**
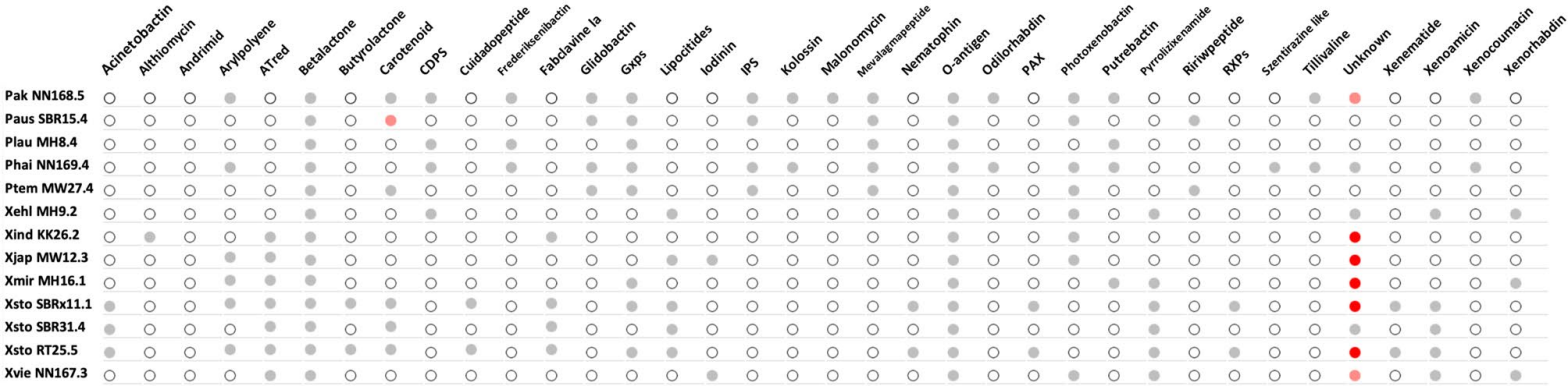
A comparative analysis of the biosynthetic gene cluster (BGCs). Different colors in the circles indicate the number of clusters: white for no cluster, gray for 1 cluster, orange for 2 clusters, and red for more than 3 clusters.

In order to identify distinctive or divergent gene clusters associated with potential antimicrobial synthesis, a comparative analysis of the strains' biosynthetic potential was conducted in relation to other strains. The findings revealed that NRPS constituted the predominant class of biosynthetic gene clusters (BGCs) in both *Xenorhabdus* and *Photorhabdus* genomes, comprising 51% of the total BGCs. While the ‘others’ and the hybrid groups displayed the second and third highest levels of enrichment and distribution, respectively. In contrast, the PKS, RiPPs, and terpene were comparatively scarce across all the genomes. Through in-silico analysis, it was observed that certain clusters were shared among multiple strains. The clusters exhibited the highest degree of sharing were those resembling known clusters responsible for betalactone production^13^, followed by gameXpeptides (Gxps) clusters^14,15^, and clusters associated with photoxenobactin^16^. Despite the extensive diversity of BGCs in all genomes, only a limited number of clusters associated with secondary metabolite production, such as acinetobactin, ATred, butyrolactone, cuidadopeptide, peptide antimicrobial-*Xenorhabdus* (PAX peptide), and rhabdopeptide/xenortide peptides (RXPs), were more prevalent only in* X. stockiae* genomes. Additionally, exclusive discovery of althiomycin, andrimid, and malonomycin clusters was found in *X. indica* strain Xind KK26.2, *P. temperata* strain Ptem MW27.4, and *P. akhurstii* strain Pak NN168.5, respectively (Fig. 3). These strains may hold the potential to serve as distinctive sources for experimental natural product discovery.

### Orphan NRPS/PKS gene clusters

**Figure 4:**
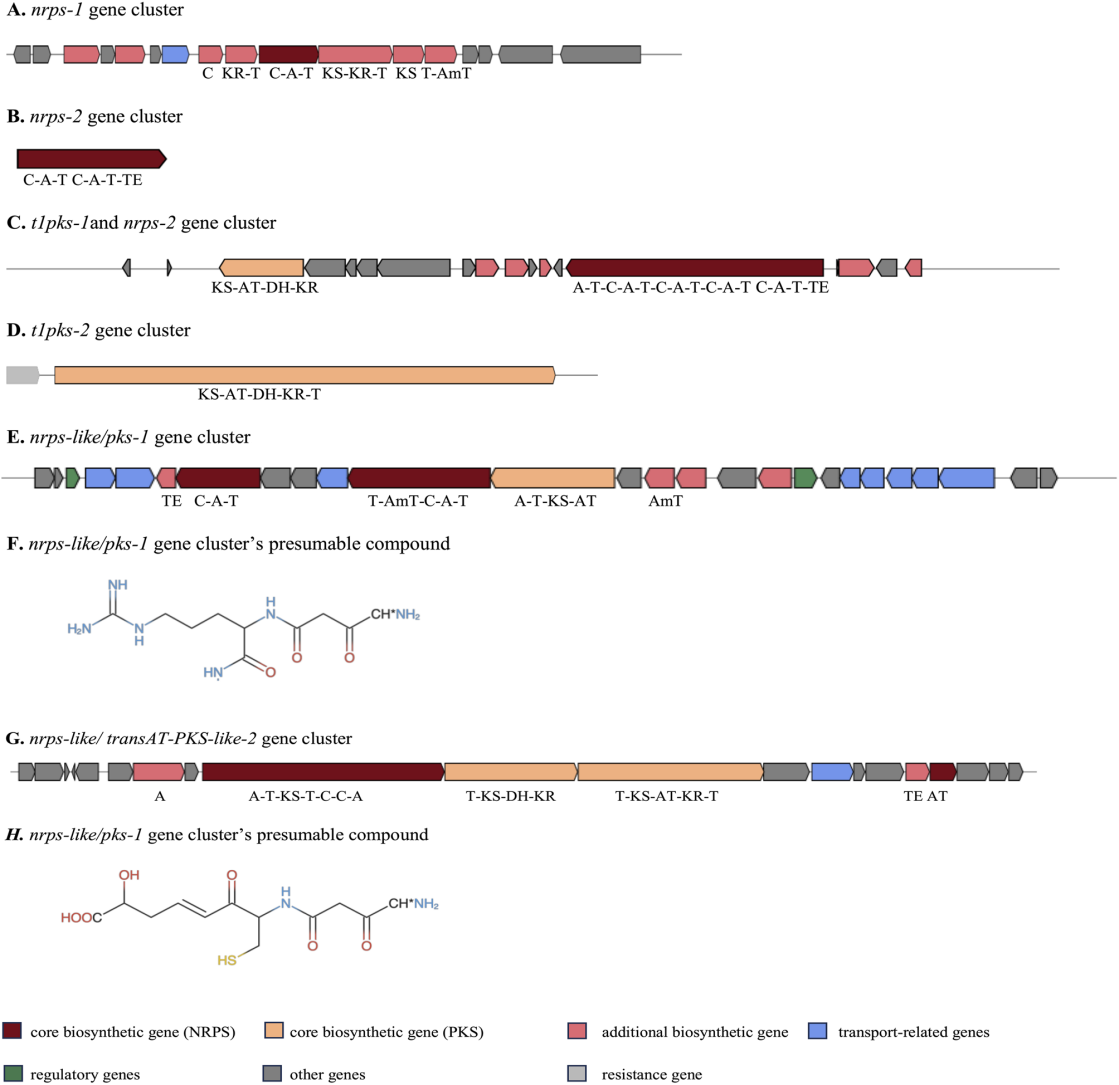
The summary of the domain composition and organization of the uncharacterized clusters. The clusters including nrps-1 gene cluster (**A**), nrps-2 gene cluster (**B**), t1pks-1and nrps-2 gene cluster (**C**), t1pks-2 gene cluster (**D**), nrps-like/pks-1 gene cluster (**E**) and its presumable compound (**F**), and nrps-like/ transAT-PKS-like-2 gene cluster (**G**) and its presumable compound (**H**).

Through the analysis of the sequence-based similarity network of BGCs, a total of 22 unknown clusters were discovered. These clusters were further characterized and classified into ten putative BGCs. Among them, three clusters were classified as NRPS clusters, two cluster belonged to the type I T1PKS category, two clusters exhibited hybrid NRPS/PKS characteristics, and three clusters were in ‘others’ group. The *nrpks-1* gene clusters were found in the region 25.1 of *P. temperata* strain Ptem MW27.4, region 3.1 of *P. hainanensis* strain Phai NN169.4, and region 24.1 of *P. akhurstii* strain Pak NN168.5 (Fig. 4A). These clusters spanned approximately 37 kb and comprised over 30 genes associated with the biosynthetic process. However, the clusters contained only a single module and showed low sequence similarities to characterized NRPS. Therefore, we were not able to predict the metabolites. The *nrpk-2* gene clusters were identified in region 3.1 of both *X. stockiae* strain Xsto RT25.5 and *X. stockiae* strain Xsto SBRx11.1 (Fig. 4B). Although, these clusters were not fully sequenced and did not yield any matches in the databases, the clusters consisted of two modules, indicating that the products were likely to be dipeptides, specifically hydrophobic-aliphatic peptides containing valine (Val)—valine (Val) as the constituent building blocks.

Within region 8.1 of the *X. stockiae* strain Xsto RT25.5 and *X. stockiae* strain Xsto SBRx11.1 genomes, we uncovered one unidentified *t1pks-1* gene clusters and one unidentified *nrpks-3* gene cluster (Fig. 4C). To distinguish these clusters, we have named them as “*plu00736*” and “*plu00747*,” respectively. The absence of matches in the MIBiG, Known ClusterBlast, and our in-house databases emphasized their uniqueness and implied the presence of two potential novel compounds in this genomic region. Moreover, the existence of a self-resistant gene underscored the significance of these genes and suggested their potential antimicrobial activity. Within the *t1pks-1* gene clusters, we hypothesized the presence of PKS_KS (Modular-KS), PKS_AT (Modular-AT), PKS_DH Modular-DH), and PKS_KR (Modular-KR) domains which having methylmalonyl-CoA as the substrate. In the NRPS cluster, the genes are predicted to encode 5 modules showing valine (Val)—leucine (Leu)—leucine (Leu)—leucine (Leu)—leucine (Leu) as the primary amino acids. In addition, the t1*pks-2* gene clusters, located in region 64.1 of *P. akhurstii* strain Pak NN168.5 and region 49.1 of *P. hainanensis* strain Phai NN169.4, displayed a similarity score of 81.37% with *t1pks-*1 from the region 8.1 of *X. stockiae*, except for the phosphopantetheine-binding protein (PP-binding) domain. These clusters could potentially represent analogues of the same compound (Fig. 4D).

Regarding the *hybrid-1* gene clusters from *nrps-like* and *t1pks-1* genes in the region 16.1 of *X. stockiae* strain Xsto RT25.5, region 16.1 of *X. stockiae* strain Xsto SBRx11.1, and region 11.1 of *X. stockiae* strain Xsto SBR31.4 spanned 57.4 kb in size and contained up to forty related genes, whose assembly line was composed of seven modules (Fig. 4E). According to the assembly line rule and the substrates of domains, the gene clusters were assumed to synthesize the product shown in Fig. 4F. From NORINE database, the structure has similar characteristics to those of a bacterial small molecule that is used worldwide as a protease inhibitor. This molecule has served as a well-established chemical model in the fields of autophagy and immunoproteasome research^17^. *Hybrid-2* gene clusters is a NRPS-like/transAT-PKS-like hybrid gene in *X. miraniensis* strain Xmir MH16.1 region 32.1, *X. vietnamensis* strain Xvei NN167.3 region 42.1, and *X. japonica* strain Xjap MW12.3 region 30.1 encoding a protein comprising five modules and one domain was predicted to incorporate cysteine (Cys) as the substrates (Fig. 4G); therefore, the products were predicted to be hexapeptides including one cysteine molecules (Fig. 4H). For the remaining clusters (data not shown), three distinct types were identified. Firstly, in the *X. miraniensis* strain Xmir MH16.1 region 23.1 and *X. indica* strain Xind KK26.2 region 22, we observed nucleotide-related clusters. In *X. japonica* strain Xjap MW12.3 region 43.1 and *X. indica* strain Xind KK26.2 region 14.1, phosphonate-related clusters were presented and within the *X. miraniensis* strain Xmir MH16.1 region 32.1, we identified lanthipeptide-class-II clusters. Unfortunately, none of these modules could be predicted from the gene clusters, and they exhibited low sequence similarities to characterized compounds. As a result, we were unable to predict the metabolites associated with these clusters.

## Discussion

### Genome mining of secondary metabolites and BGCs distribution

The average size of the 13 *Xenorhabdus* and *Photorhabdus* genomes from Thailand was approximately 4.6 Mb with an average of 24 biosynthetic gene clusters (BGCs). The count of BGCs is deemed high when compared to other genera in the same family^18^. A greater number of BGCs indicated a greater potential to produce bioactive substances and secondary metabolites^19^. Furthermore, the outcomes from analyzing the pan-genome revealed certain resemblances in the genomic sequences of these two bacterial genera. Both bacteria have a set of core genes which are commonly found across species. These core genes are essential for basic cellular functions and are relatively conserved in terms of sequence and function^20^. Nevertheless, there are noteworthy distinctions observed between the two genera. Each genus harbors a unique collection of genes specific to its genus and species, which play a pivotal role in shaping their distinct characteristics and lifestyles^13^. These genes reflect their adaptation to diverse ecological niches, encompassing regulatory elements as well as the production of secondary metabolites such as antibiotics and toxins, which are uncommonly encountered in other organisms^21^. Likewise, the genes associated with the biosynthesis of secondary metabolites can demonstrate considerable variability and diversity among different species and strains. Such variations play a significant role in generating a wide array of distinct bioactive compounds.

### Sequence-based similarity network of BGCs

The analysis highlighted the diversity of BGCs within the genus, indicating the biosynthetic potential of these symbiotic bacterium, including the possibility of biodiscovery of novel secondary compounds from the isolates. A total of 160 biosynthetic clusters with likeness to recognized BGCs contribute to the bacteria's role in pathogenesis, defense against competing microorganisms, and the establishment of the nematode-bacteria symbiosis which has been found to exhibit significant inhibitory effects against various microorganisms, including bacteria, fungi, and some parasites^7,9,22^. Among the strains examined in this study, *X. indica* strain Xind KK26.2 and *X. vietnamensis* strain Xvei NN167.3 prominently exhibit the highest prevalence of the secondary metabolite biosynthetic gene clusters (smBGCs), suggesting their potential for uncovering novel bioactive secondary metabolites. This hypothesis is further supported by antiSMASH analyses, revealing that both *X. indica* strain Xind KK26.2 and *X. vietnamensis* strain Xvei NN167.3 strains possess an enrichment of over 28 BGCs. As a result, they are attractive candidates for the development of novel antibiotics or antimicrobial medicines. However, additional research is required to completely comprehend its prospective uses and enhance its effectiveness. During the subsequent analysis, the refined genomes exhibited similarities by harboring gene clusters associated with known clusters including xenocoumacin^23–25^, szentirazine^26^, frederiksenibactin^27^, photoxenobactin^16^, tilivalline^28^, putrebactin^29^, odilorhabdin^30^, o-antigen^31^, gameXpeptides (Gxps)^14,15^, mevalagmapeptide^15^, betalactone^13^, kolossin^32^, isopropylstilbene (IPS)^33^, CDPS^34^, malonomycin^35^, acinetobactin^36^, aryl polyene^37^, ririwpeptide^15^, andrimid^38,39^, pyrrolizixenamide^40^, xenoamicin^41^, xenorhabdin^42^, lipocitides^13^, fabclavine^43^, Acyltransferase Red (ATred)^44^, phenazine^45^, cuidadopeptide^26,46^, althiomycin^47^, PAX (peptide-antimicrobial-*Xenorhabdus*)^48–50^, xenematide^7^, nematophin^51^, rhabdopeptide (RXPs)^52^, glidobactin^53^, and butyrolactone^54^.

### Comparison of the biosynthetic gene clusters

The findings of this study exhibited remarkable similarities to the analysis conducted by Shi and colleagues in 2022, where NRPS were identified as the most abundant class of biosynthetic gene clusters (BGCs) in both *Xenorhabdus* and *Photorhabdus* genomes. The high prevalence of NRPS BGCs suggests that their products could play significant ecological roles^13^. Additionally, the others group emerged as the second-largest class of BGCs, with its products potentially aiding bacteria in performing specific ecological functions^13^. The hybrid class of PKS/NRPS shows a slightly enrichment and distribution, while PKS, RiPPs, and terpene BGCs are relatively scarce across all genomes compared to other types. Moreover, in alignment with previous studies^13^, betalactone clusters were identified as the predominant cluster type followed by gameXpeptides and photoxenobactin biosynthetic clusters. The products of betalactone and Gxps clusters have been identified to play a role in insect immune suppression, while photoxenobactin has been found to exhibit insecticidal properties^13^. In addition to the mentioned clusters, the remaining clusters displayed similarities to various types of bioactive compounds with antibacterial, insecticidal, antiprotozoal, antifungal, antiparasitic properties, as well as broad-spectrum compounds like fabclavine^43^.

### Orphan NRPS/PKS gene clusters

With regards to the orphan clusters, all clusters except the T1PSK type were found to encompass genes associated with transcription regulation and transport. Previous studies have emphasized the significance of these transcription regulation and transport mechanisms in the biosynthesis of antibiotics like oleandomycin^55^ and spiramycin^56^. By integrating comparative genomics with sequence-based similarity network analyses, researchers have gained valuable insights into the genetic and BGCs (biosynthetic gene clusters) diversity present within and between *Xenorhabdus* spp. and *Photorhabdus* spp. These findings contribute to our comprehension of the distinctive or divergent gene clusters associated with their potential capacity for antimicrobial synthesis.

## Methods

### Entomopathogenic bacterial strains

Thirteen isolates of symbiotic bacteria were isolated from entomopathogenic nematodes from Thailand. Eight isolates of *Xenorhabdus* and 5 isolates of *Photorhabdus* were previously identified with *recA* sequence^57–62^ (Table 2).

**Table 2:** List of 13 isolates of *Xenorhabdus* and *Photorhabdus* bacteria used in this study.

Strain number	Bacteria species	Entomopathogenic nematode host
Xjap MW12.3	*X. japonica*	*Steinernema kushidai*
Xehl MH9.2	*X. ehlersii*	*S. scarabaei*
Xmir MH16.1	*X. miraniensis*	*S. websteiri*
Xind KK26.2	*X. indica*	*Steinernema* sp.
Xvie NN167.3	*X. vietnamensis*	Unidentified
Xsto SBRx11.1	*X. stockiae*	Unidentified
Xsto SBR31.4	*X. stockiae*	*S. surkhetense*
Xsto RT25.5	*X. stockiae*	Unidentified
Ptem MW27.4	*P. temperata*	*H. zealandica*
Plau MH8.4	*P. laumondii*	*H*. sp. SGmg3
Pak NN168.5	*P. akhurstii*	Unidentified
Phai NN169. 4	*P. hainanensis*	Unidentified
Paus SBR15.4	*P. australis*	*H. indica*

### Genome sequencing and annotation

To initiate the experiment, a single colony of each strain was inoculated in 5 ml of Luria–Bertani broth^40^ and incubated at 28 °C with agitation overnight. Genomic DNA was isolated using the DNeasy kit (Qiagen, Hilden, Germany). For next-generation sequencing, mate pair libraries were prepared using the Nextera XT DNA Library Preparation Kit (Illumina, San Diego, CA, USA) following the manufacturer's instructions. All libraries underwent sequencing in 250 bp paired read runs on the Illumina MiSeq platform. The resulting reads were subjected to quality trimming using Sickle v.1.33^63^, discarding any trimmed reads shorter than 125 bp. Subsequently, genome assembly was performed using SPAdes v. 3.10.1^64^ with the following parameters: --cov-cutoff auto, --careful in paired-end mode plus mate pairs, and k-mer lengths of 21, 33, 55, 77, 81, and 91. Genome annotation was conducted using Prokka v. 1.12^65^ with the following parameters: --usegenus --genus GENUS –addgenes --evalue 0.0001 --rfam --kingdom Bacteria --gcode 11 --gram --mincontiglen 200.

### Bioinformatics analysis

The pan and core genome analysis of annotated genomes herein mainly followed the anvi’o 7.1 pan genomic workflow^66,67^. Briefly, an anvi’o genomes-storage-db was generated using the program anvi-gen-genomes-storage. After that, an anvi’o pan-db was performed using the program anvi-pan-genome. Lastly, the results were displayed in anvi’o interactive interface using the program anvi-display-pan^67^. After that, the 13 genome sequences, annotated in the GenBank format, were analyzed for biosynthetic gene cluster (BGC) identification using the antiSMASH (antibiotics and secondary metabolite analysis shell) 6.1.1 pipeline. The detection strictness was set to “relaxed,” and the ClusterBlast, Cluster Pfam analysis, and Pfam-based GO options were enabled for the antiSMASH analysis. After the initial BGC types were assigned by antiSMASH, a manual inspection and reclassification process took place, ensuring accurate classification. A summary of the annotated BGCs was then generated. Subsequently, the antiSMASH job IDs were utilized to explore and classify the BGCs using the biosynthetic gene cluster family's database, BiG-FAM 1.0.0^68^, providing preliminary insights. To refine the analysis, BiG-SCAPE 1.0.0^68^ was employed with a cutoff value of 0.65. The resulting gene cluster families (GCFs) underwent a thorough manual double-check using an interactive network, and necessary corrections were applied. Finally, the conclusive outcomes were visualized as a network using Cytoscape 3.10.

### Supplementary Information

Supplementary Table S1.

Supplementary Table S2.

Supplementary Table S3.

Supplementary Table S4.

Supplementary Table S5.

## Data Availability

The genome sequence of *Xenorhabdus* and *Photorhabdus* data that support the findings of this study are available in NCBI GenBank database under accession SAMN36278238 to SAMN36278250. All other data generated or analysed in this study are available within the article and its supplementary information. Supporting Information files are published exactly as provided, and are not modified or copyedited.
