# Supplementary material for: Genome mining reveals novel biosynthetic gene clusters in entomopathogenic bacteria
Source: Sci Rep. 2023 Nov 25;13:20764. doi: 10.1038/s41598-023-47121-9 (PMC10676414; doi:10.1038/s41598-023-47121-9)
Supplement: Supplementary file 1 — Supplementary Table S1. [file 41598_2023_47121_MOESM1_ESM.pdf]

Supplementary Table S1. The strains employed in bioinformatics analysis

**Accession**	**BioProject**	**Sample name**	**Organism name**	**Release date**
SAMN36278250	PRJNA990961	Xvie NN167.3	*Xenorhabdus vietnamensis*	7/4/2023
SAMN36278249	PRJNA990961	Xsto RT25.5	*Xenorhabdus stockiae*	7/4/2023
SAMN36278248	PRJNA990961	Xsto SBR31.4	*Xenorhabdus stockiae*	7/4/2023
SAMN36278247	PRJNA990961	Xsto SBRx11.1	*Xenorhabdus stockiae*	7/4/2023
SAMN36278246	PRJNA990961	Xmir MH16.1	*Xenorhabdus miraniensis*	7/4/2023
SAMN36278245	PRJNA990961	Xjap MW12.3	*Xenorhabdus japonica*	7/4/2023
SAMN36278244	PRJNA990961	Xind KK26.2	*Xenorhabdus indica*	7/4/2023
SAMN36278243	PRJNA990961	Xehl MH9.2	*Xenorhabdus ehlersii*	7/4/2023
SAMN36278242	PRJNA990961	Ptem MW27.4	*Photorhabdus temperata*	7/4/2023
SAMN36278241	PRJNA990961	Phai NN169.4	*Photorhabdus hainanensis*	7/4/2023
SAMN36278240	PRJNA990961	Plau MH8.4	*Photorhabdus laumondii*	7/4/2023
SAMN36278239	PRJNA990961	Pak NN168.5	*Photorhabdus akhurstii*	7/4/2023
SAMN36278238	PRJNA990961	Paus SBR15.4	*Photorhabdus australis*	7/4/2023
