# Supplementary material for: Genome mining reveals novel biosynthetic gene clusters in entomopathogenic bacteria
Source: Sci Rep. 2023 Nov 25;13:20764. doi: 10.1038/s41598-023-47121-9 (PMC10676414; doi:10.1038/s41598-023-47121-9)
Supplement: Supplementary file 2 — Supplementary Table S2. [file 41598_2023_47121_MOESM2_ESM.pdf]

Supplementary Table S2. Statistical analysis of contigs in *Xenorhabdus* and *Photorhabdus* genome sequencing

**Contigs Stats**	Pak NN168.5	Paus SBR15.4	Phai NN169.4	Plau MH8.4	Ptem MW27.4	Xehl MH9.2	Xind KK26.2	Xjap MW12.3	Xmir MH16.1	Xsto RT25.5	Xsto SBR31.4	Xsto SBRx11.1	Xvie NN167.3
Total Length	5,629,344	4,699,665	5,260,969	4,937,123	5,247,567	3,916,271	4,506,415	3,495,382	4,378,409	4,509,455	4,496,578	4,509,288	4,654,759
Num Contigs	79	87	50	110	161	47	95	52	80	132	52	132	83
Num Contigs > 100 kb	18	12	20	13	9	14	14	13	16	6	17	6	15
Num Contigs > 50 kb	32	32	28	33	29	21	38	24	24	34	29	34	33
Num Contigs > 20 kb	50	55	42	65	72	33	50	37	39	69	38	69	52
Num Contigs > 10 kb	55	63	44	83	100	39	66	40	48	96	44	96	69
Num Contigs > 5 kb	66	73	49	94	129	44	84	45	61	111	48	111	75
Num Contigs > 2.5 kb	79	87	50	110	161	47	95	52	80	132	52	132	83
Longest Contig	429,773	250,279	458,435	289,965	320,441	604,063	233,439	378,067	319,343	185,193	283,943	185,483	300,582
Shortest Contig	2,679	2,612	3,974	2,707	2,537	3,204	2,595	2,766	2,608	2,687	3,386	2,687	2,501
Num Genes (prodigal)	4,805	4,152	4,337	4,351	4,582	3,383	3,907	3,008	3,739	3,921	3,832	3,919	3,947
L50	11	14	10	18	22	7	17	9	10	26	10	25	15
L75	23	29	19	37	50	15	32	19	20	49	20	49	29
L90	38	47	29	61	83	24	49	29	34	76	31	76	46
N50	161,766	91,246	168,036	83,047	66,115	176,708	93,815	128,329	162,938	62,766	154,677	64,259	101,518
N75	79,874	57,975	107,734	46,313	32,744	92,215	62,268	65,480	73,980	34,367	81,111	34,367	56,847
N90	42,852	30,276	46,728	21,575	14,617	37,737	25,029	40,821	30,364	17,541	47,989	17,541	31,150
