# Supplementary material for: Genome mining reveals novel biosynthetic gene clusters in entomopathogenic bacteria
Source: Sci Rep. 2023 Nov 25;13:20764. doi: 10.1038/s41598-023-47121-9 (PMC10676414; doi:10.1038/s41598-023-47121-9)
Supplement: Supplementary file 5 — Supplementary Table S5. [file 41598_2023_47121_MOESM5_ESM.pdf]

Supplementary Table S5. An overview of annotated BGCs subjected to refinement by BiG-SCAPE and our in-house database

NO	XP isolates	Region	Clusters type	Predicted compounds
1	*P. hainaensis*	Phai NN169.4 region 1.1	NRP+Polyketide	Xenocoumacin
2	*P. hainaensis*	Phai NN169.4 region 1.2	NRPS	Szentirazine like
3	*P. hainaensis*	Phai NN169.4 region 10.1	NRPS	Frederiksenibactin
4	*P. hainaensis*	Phai NN169.4 region 11.2	NRP+Polyketide	Photoxenobactin
5	*P. hainaensis*	Phai NN169.4 region 13.1	NRPS	tillivaline
6	*P. hainaensis*	Phai NN169.4 region 16.1	NRPS.independent.siderophore	Putrebactin
7	*P. hainaensis*	Phai NN169.4 region 17.1	NRPS	Odilorhabdin
8	*P. hainaensis*	Phai NN169.4 region 18.1	RiPPs	O-antigen
9	*P. hainaensis*	Phai NN169.4 region 2.1	NRPS	Gxps
10	*P. hainaensis*	Phai NN169.4 region 25.1	NRPS	Mevalagmapeptide
11	*P. hainaensis*	Phai NN169.4 region 3.1	NRPS	Unknown
12	*P. hainaensis*	Phai NN169.4 region 3.2	Others	Betalactone
13	*P. hainaensis*	Phai NN169.4 region 34.1	RiPPs	O-antigen
14	*P. hainaensis*	Phai NN169.4 region 4.1	NRPS	Kolossin
15	*P. hainaensis*	Phai NN169.4 region 49.1	PKS	Unknown
16	*P. hainaensis*	Phai NN169.4 region 5.1	NRP+Polyketide	Glidobactin
17	*P. hainaensis*	Phai NN169.4 region 6.1	Others	Arylpolyene
18	*P. hainaensis*	Phai NN169.4 region 6.2	PKS	Isopropylstilbene (IPS)
19	*P. hainaensis*	Phai NN169.4 region 7.1	Others	CDPS
20	*P. hainaensis*	Phai NN169.4 region 9.1	NRPS	Malonomycin
21	*P. laumondii*	PLau MH8.4 region 1.1	NRP+Polyketide	Glidobactin
22	*P. laumondii*	PLau MH8.4 region 10.1	Others	CDPS
23	*P. laumondii*	PLau MH8.4 region 11.1	NRPS	Gxps
24	*P. laumondii*	PLau MH8.4 region 19.2	RiPPs	O-antigen
25	*P. laumondii*	PLau MH8.4 region 3.1	NRP+Polyketide	Photoxenobactin
26	*P. laumondii*	PLau MH8.4 region 38.1	RiPPs	O-antigen
27	*P. laumondii*	PLau MH8.4 region 4.1	NRPS	Frederiksenibactin
28	*P. laumondii*	PLau MH8.4 region 5.1	NRPS	Mevalagmapeptide
29	*P. laumondii*	PLau MH8.4 region 9.1	Others	Betalactone
30	*P. akhurstii*	Pak NN168.5 region 28.1	Terpene	Carotenoid
31	*P. akhurstii*	Pak NN168.5 region 1.1	NRP+Polyketide	Xenocoumacin
32	*P. akhurstii*	Pak NN168.5 region 12.1	Others	CDPS
33	*P. akhurstii*	Pak NN168.5 region 13.1	NRPS	Gxps
34	*P. akhurstii*	Pak NN168.5 region 15.1	NRPS	Odilorhabdin
35	*P. akhurstii*	Pak NN168.5 region 16.1	Others	Betalactone
36	*P. akhurstii*	Pak NN168.5 region 2.1	NRP+Polyketide	Photoxenobactin
37	*P. akhurstii*	Pak NN168.5 region 24.1	NRPS	Malonomycin
38	*P. akhurstii*	Pak NN168.5 region 3.1	NRP+Polyketide	Glidobactin
39	*P. akhurstii*	Pak NN168.5 region 3.2	RiPPs	O-antigen
40	*P. akhurstii*	Pak NN168.5 region 41.1	NRPS	Unknown
41	*P. akhurstii*	Pak NN168.5 region 5.1	NRPS.independent.siderophore	Putrebactin
42	*P. akhurstii*	Pak NN168.5 region 5.2	NRPS	Tillivaline
43	*P. akhurstii*	Pak NN168.5 region 6.1	NRPS	Kolossin
44	*P. akhurstii*	Pak NN168.5 region 6.2	NRPS	Frederiksenibactin
45	*P. akhurstii*	Pak NN168.5 region 64.1	PKS	Unknown
46	*P. akhurstii*	Pak NN168.5 region 7.1	Others	Arylpolyene
47	*P. akhurstii*	Pak NN168.5 region 7.2	PKS	Isopropylstilbene (IPS)
48	*P. akhurstii*	Pak NN168.5 region 9.1	NRPS	Mevalagmapeptide
49	*P. australis*	Phai NN169.4 region 1.3	Terpene	Carotenoid
50	*P. australis*	Plau MH8.4 region 79.1	Terpene	Carotenoid

Supplementary Table S5. An overview of annotated BGCs subjected to refinement by BiG-SCAPE and our in-house database (Cont.)

NO	XP isolates	Region	Clusters type	Predicted compounds
51	*P. australis*	Paus SBR 15.4 region 51.1	NRPS	Mevalagmapeptide
52	*P. australis*	Paus SBR15.4 region 11.1	PKS	Isopropylstilbene (IPS)
53	*P. australis*	Paus SBR15.4 region 13.1	NRP+Polyketide	Glidobactin
54	*P. australis*	Paus SBR15.4 region 25.1	NRPS	Ririwpeptide
55	*P. australis*	Paus SBR15.4 region 34.1	NRP+Polyketide	Photoxenobactin
56	*P. australis*	Paus SBR15.4 region 46.1	NRPS	Gxps
57	*P. australis*	Paus SBR15.4 region 7.1	Others	Betalactone
58	*P. australis*	Paus SBR15.4 region 8.1	RiPPs	O-antigen
59	*P. temperata*	Ptem MW27.4 region 2.2	Others	Betalactone
60	*P. temperata*	Ptem MW27.4 region 20.1	NRP+Polyketide	Andrimid
61	*P. temperata*	Ptem MW27.4 region 25.1	NRPS	Unknown
62	*P. temperata*	Ptem MW27.4 region 4.1	PKS	Isopropylstilbene (IPS)
63	*P. temperata*	Ptem MW27.4 region 40.1	Others	CDPS
64	*P. temperata*	Ptem MW27.4 region 41.1	NRPS	Szentirazine like
65	*P. temperata*	Ptem MW27.4 region 47.1	NRPS	Gxps
66	*P. temperata*	Ptem MW27.4 region 58.1	Others	Arylpolyene
67	*P. temperata*	Ptem MW27.4 region 64.1	RiPPs	O-antigen
68	*X. ehlersii*	Xehl MH9.2 region 1.1	Others	Betalactone
69	*X. ehlersii*	Xehl MH9.2 region 1.2	NRPS	Unknown_acinetobactin
70	*X. ehlersii*	Xehl MH9.2 region 17.1	NRPS	Pyrrolizixenamide
71	*X. ehlersii*	Xehl MH9.2 region 2.1	RiPPs	O-antigen
72	*X. ehlersii*	Xehl MH9.2 region 3.2	NRPS	Xenoamicin
73	*X. ehlersii*	Xehl MH9.2 region 5.1	NRP+Polyketide	Photoxenobactin
74	*X. ehlersii*	Xehl MH9.2 region 6.1	NRPS	Xenorhabdin
75	*X. ehlersii*	Xehl MH9.2 region 8.1	Others	CDPS
76	*X. ehlersii*	Xehl MH9.2 region 9.1	NRPS	Lipocitides
77	*X. indica*	Xin KK26.2 region 1.1	Others	Fabclavine la
78	*X. indica*	Xin KK26.2 region 14.1	Others	Unknown
79	*X. indica*	Xin KK26.2 region 2.1	NRPS	Unknown_acinetobactin
80	*X. indica*	Xin KK26.2 region 22.1	Others	Unknown
81	*X. indica*	Xin KK26.2 region 28.1	NRPS	ATred
82	*X. indica*	Xin KK26.2 region 34.1	RiPPs	O-antigen
83	*X. indica*	Xin KK26.2 region 44.1	NRP+Polyketide	Althiomycin
84	*X. indica*	Xin KK26.2 region 5.1	Others	Betalactone
85	*X. indica*	Xin KK26.2 region 7.1	NRP+Polyketide	Photoxenobactin
86	*X. japonica*	Xjap MW12.3 region 1.2	NRP+Polyketide	Photoxenobactin
87	*X. japonica*	Xjap MW12.3 region 16.1	NRPS	ATred
88	*X. japonica*	Xjap MW12.3 region 17.1	RiPPs	O-antigen
89	*X. japonica*	Xjap MW12.3 region 2.1	NRPS	Unknown_acinetobactin
90	*X. japonica*	Xjap MW12.3 region 24.1	Others	Iodinin (phenazine)
91	*X. japonica*	Xjap MW12.3 region 3.1	Others	Arylpolyene
92	*X. japonica*	Xjap MW12.3 region 30.1	NRP+Polyketide	Unknown
93	*X. japonica*	Xjap MW12.3 region 38.1	NRPS	Fragment
94	*X. japonica*	Xjap MW12.3 region 43.1	Others	Unknown
95	*X. japonica*	Xjap MW12.3 region 5.1	NRPS	Lipocitides
96	*X. japonica*	Xjap MW12.3 region 6.1	Others	Betalactone
97	*X. miraniensis*	Xmir MH16.1 region 1.1	RiPPs	O-antigen
98	*X. miraniensis*	Xmir MH16.1 region 1.3	NRPS.independent.siderophore	Putrebactin
99	*X. miraniensis*	Xmir MH16.1 region 16.2	NRPS	Pyrrolizixenamide
100	*X. miraniensis*	Xmir MH16.1 region 2.1	NRPS	Unknown_acinetobactin

Supplementary Table S5. An overview of annotated BGCs subjected to refinement by BiG-SCAPE and our in-house database (Cont.)

NO	XP isolates	Region	Clusters type	Predicted compounds
101	*X. miraniensis*	Xmir MH16.1 region 23.1	Others	Unknown
102	*X. miraniensis*	Xmir MH16.1 region 26.1	NRPS	Gxps
103	*X. miraniensis*	Xmir MH16.1 region 3.1	NRPS	Xenorhabdin
104	*X. miraniensis*	Xmir MH16.1 region 32.1	lanthipeptide.class.II	Unknown
105	*X. miraniensis*	Xmir MH16.1 region 32.1	NRP+Polyketide	Unknown
106	*X. miraniensis*	Xmir MH16.1 region 4.1	Others	Arylpolyene
107	*X. miraniensis*	Xmir MH16.1 region 4.2	NRPS	ATred
108	*X. miraniensis*	Xmir MH16.1 region 6.1	Others	Betalactone
109	*X. stockiae*	Xsto SBR31.4 region 5.1	NRPS	Pyrrolizixenamide
110	*X. stockiae*	Xsto SBR31.4 region 5.1	NRPS	ATred
111	*X. stockiae*	Xsto SBR31.4 region 25.1	NRPS	acinetobactin (NRP-metallophore)
112	*X. stockiae*	Xsto SBR31.4 region 26.1	NRPS	lipocitides
113	*X. stockiae*	Xsto SBR31.4 region 35.1	Terpene	Carotenoid
114	*X. stockiae*	Xsto SBR31.4 region 4.1	NRPS	Xenoamicin
115	*X. stockiae*	Xsto SBR 31.4 region 3.2	Others	Betalactone
116	*X. stockiae*	Xsto SBR31.4 region 11.1	NRP+Polyketide	Unknown
117	*X. stockiae*	Xsto SBR31.4 region 31.1	NRPS	ATred
118	*X. stockiae*	Xsto SBR31.4 region 6.1	Others	Fabclavine la
119	*X. stockiae*	Xsto SBR31.4 region 13.1	RiPPs	O-antigen
120	*X. stockiae*	Xsto RT25.5 region 18.1	Terpene	Carotenoid
121	*X. stockiae*	Xsto RT25.5 region 15.1	RiPPs	O-antigen
122	*X. stockiae*	Xsto RT25.5 region 16.1	NRP+Polyketide	Unknown
123	*X. stockiae*	Xsto RT25.5 region 17.1	Others	Arylpolyene
124	*X. stockiae*	Xsto RT25.5 region 18.1	NRPS	Cuidadopeptide
125	*X. stockiae*	Xsto RT25.5 region 20.1	Others	Betalactone
126	*X. stockiae*	Xsto RT25.5 region 24.1	NRPS	Pyrrolizixenamide
127	*X. stockiae*	Xsto RT25.5 region 24.1	NRPS	ATred
128	*X. stockiae*	Xsto RT25.5 region 3.1	Others	Fabclavine la
129	*X. stockiae*	Xsto RT25.5 region 34.1	NRPS	ATred
130	*X. stockiae*	Xsto RT25.5 region 37.1	NRPS	Acinetobactin (NRP-metallophore)
131	*X. stockiae*	Xsto RT25.5 region 4.1	NRPS	Xenoamicin
132	*X. stockiae*	Xsto RT25.5 region 49.1	NRPS	Lipocitides
133	*X. stockiae*	Xsto RT25.5 region 56.1	Others	Butyrolactone
134	*X. stockiae*	Xsto RT25.5 region 58.1	NRPS	Fragment
135	*X. stockiae*	Xsto RT25.5 region 59.1	NRPS	Fragment
136	*X. stockiae*	Xsto RT25.5 region 61.1	NRPS	Xenematide
137	*X. stockiae*	Xsto RT25.5 region 8.1	PKS	Unknown
138	*X. stockiae*	Xsto RT25.5 region 8.1	NRPS	Unknown
139	*X. stockiae*	Xsto RT25.5 region 8.1	NRPS	Nematophin, Rhabdopeptide (RXPs), GameXpeptide
140	*X. stockiae*	Xsto RT25.5 region 81.1	NRPS	Fragment
141	*X. stockiae*	Xsto RT25.5 region 85.1	NRPS	PAX (with glutamine)
142	*X. stockiae*	Xsto RT25.5 region 90.1	NRPS	Fragment
143	*X. stockiae*	Xsto RT25.5 region 3.1	NRPS	Unknown
144	*X. stockiae*	Xsto SBRx11.1 region 18.1	Terpene	Carotenoid
145	*X. stockiae*	Xsto SBRx11.1 region 10.1	Others	Betalactone
146	*X. stockiae*	Xsto SBRx11.1 region 15.1	RiPPs	O-antigen
147	*X. stockiae*	Xsto SBRx11.1 region 16.1	NRP+Polyketide	Unknown
148	*X. stockiae*	Xsto SBRx11.1 region 17.1	Others	Aryl polyene
149	*X. stockiae*	Xsto SBRx11.1 region 18.1	NRPS	Cuidadopeptide
150	*X. stockiae*	Xsto SBRx11.1 region 24.1	NRPS	Pyrrolizixenamide

Supplementary Table S5. An overview of annotated BGCs subjected to refinement by BiG-SCAPE and our in-house database (Cont.)

NO	XP isolates	Region	Clusters type	Predicted compounds
151	*X. stockiae*	Xsto SBRx11.1 region 24.1	NRPS	ATred
152	*X. stockiae*	Xsto SBRx11.1 region 3.1	PKS/NRPS	Unknown
153	*X. stockiae*	Xsto SBRx11.1 region 3.1	Others	Fabclavine la
154	*X. stockiae*	Xsto SBRx11.1 region 34.1	NRPS	ATred
155	*X. stockiae*	Xsto SBRx11.1 region 37.1	NRPS	Acinetobactin (NRP-metallophore)
156	*X. stockiae*	Xsto SBRx11.1 region 4.1	NRPS	Xenoamicin
157	*X. stockiae*	Xsto SBRx11.1 region 49.1	NRPS	lipocitides
158	*X. stockiae*	Xsto SBRx11.1 region 56.1	Others	butyrolactone
159	*X. stockiae*	Xsto SBRx11.1 region 58.1	NRPS	Fragment
160	*X. stockiae*	Xsto SBRx11.1 region 59.1	NRPS	Fragment
161	*X. stockiae*	Xsto SBRx11.1 region 61.1	NRPS	Xenematide
162	*X. stockiae*	Xsto SBRx11.1 region 8.1	PKS	Unknown
163	*X. stockiae*	Xsto SBRx11.1 region 8.1	NRPS	Unknown
164	*X. stockiae*	Xsto SBRx11.1 region 8.1	NRPS	Nematophin, Rhabdopeptide (RXPs), GameXpeptide
165	*X. stockiae*	Xsto SBRx11.1 region 81.1	NRPS	Fragment
166	*X. stockiae*	Xsto SBRx11.1 region 85.1	NRPS	PAX (with glutamine)
167	*X. stockiae*	Xsto SBRx11.1 region 90.1	NRPS	Fragment
168	*X. vietnamensis*	Xvei NN167.3 region 1.1	RiPPs	O-antigen
169	*X. vietnamensis*	Xvei NN167.3 region 10.1	Others	Betalactone
170	*X. vietnamensis*	Xvei NN167.3 region 12.1	Others	Iodinin (phenazine)
171	*X. vietnamensis*	Xvei NN167.3 region 15.1	NRPS	Unknown_acinetobactin
172	*X. vietnamensis*	Xvei NN167.3 region 2.1	NRPS	Xenorhabdin
173	*X. vietnamensis*	Xvei NN167.3 region 22.1	NRP+Polyketide	Photoxenobactin
174	*X. vietnamensis*	Xvei NN167.3 region 24.1	NRPS	Xenoamicin
175	*X. vietnamensis*	Xvei NN167.3 region 35.1	NRPS	Pyrrolizixenamide
176	*X. vietnamensis*	Xvei NN167.3 region 42.1	NRP+Polyketide	Unknown
177	*X. vietnamensis*	Xvei NN167.3 region 5.1	NRPS	ATred
178	*X. vietnamensis*	Xvei NN167.3 region 55.1	NRPS	Fragment
